# Multidimensional Sleep Health: Concepts, Advances, and Implications for Research and Intervention

**DOI:** 10.1093/geroni/igab046.1306

**Published:** 2021-12-17

**Authors:** Joon Chung, Matthew Goodman, Tianyi Huang, Suzanne Bertisch, Susan Redline

**Affiliations:** 1 Brigham and Women's Hospital, Harvard Medical School, Boston, Massachusetts, United States; 2 Brigham and Women's Hospital, Boston, Massachusetts, United States; 3 Harvard Medical School, Boston, Massachusetts, United States

## Abstract

Sleep is a complex process, sensitive to aging, with theoretical and evidentiary basis for influence on multiple health outcomes. Recent scholarship has argued for a ‘multi-dimensional’ approach to sleep health, that is, a recognition that healthy sleep consists of more than its quantity (duration) and is more than the absence of sleep disorders. This new conception of sleep health acknowledges sleep’s complexity yet presents challenges for methodological treatment. How do we operationalize/analyze multiple dimensions of sleep, some of which are correlated due to physiological reasons, common measurement tools, or sensitivity to common stressors? Is it sensible to talk about ‘sleep health’ as a single, composite entity with multiple components, akin to a dietary pattern rather than a collection of individual nutrients? Exemplar data from a racial-ethnic disparities project in aging adults suggest the utility of a composite approach, and the value of considering inter-correlations among sleep metrics.

